# Data sharing, management, use, and reuse: Practices and perceptions of scientists worldwide

**DOI:** 10.1371/journal.pone.0229003

**Published:** 2020-03-11

**Authors:** Carol Tenopir, Natalie M. Rice, Suzie Allard, Lynn Baird, Josh Borycz, Lisa Christian, Bruce Grant, Robert Olendorf, Robert J. Sandusky

**Affiliations:** 1 School of Information Sciences, University of Tennessee, Knoxville, Tennessee, United States of America; 2 Center for Information and Communication Studies, University of Tennessee, Knoxville, Tennessee, United States of America; 3 University of Idaho Libraries, University of Idaho, Moscow, Idaho, United States of America; 4 College of Communication and Information, University of Tennessee, Knoxville, Tennessee, United States of America; 5 Departments of Biology and Environmental Science and Sustainability, Widener University, Chester, Pennsylvania, United States of America; 6 North Carolina State University Libraries, North Carolina State University, Raleigh, North Carolina, United States of America; 7 UIC University Library, University of Illinois at Chicago, Chicago, Illinois, United States of America; Universitat de Barcelona, SPAIN

## Abstract

**Background:**

With data becoming a centerpiece of modern scientific discovery, data sharing by scientists is now a crucial element of scientific progress. This article aims to provide an in-depth examination of the practices and perceptions of data management, including data storage, data sharing, and data use and reuse by scientists around the world.

**Methods:**

The Usability and Assessment Working Group of DataONE, an NSF-funded environmental cyberinfrastructure project, distributed a survey to a multinational and multidisciplinary sample of scientific researchers in a two-waves approach in 2017–2018. We focused our analysis on examining the differences across age groups, sub-disciplines of science, and sectors of employment.

**Findings:**

Most respondents displayed what we describe as high and mediocre risk data practices by storing their data on their personal computer, departmental servers or USB drives. Respondents appeared to be satisfied with short-term storage solutions; however, only half of them are satisfied with available mechanisms for storing data beyond the life of the process. Data sharing and data reuse were viewed positively: over 85% of respondents admitted they would be willing to share their data with others and said they would use data collected by others if it could be easily accessed. A vast majority of respondents felt that the lack of access to data generated by other researchers or institutions was a major impediment to progress in science at large, yet only about a half thought that it restricted their own ability to answer scientific questions. Although attitudes towards data sharing and data use and reuse are mostly positive, practice does not always support data storage, sharing, and future reuse. Assistance through data managers or data librarians, readily available data repositories for both long-term and short-term storage, and educational programs for both awareness and to help engender good data practices are clearly needed.

## Introduction

Science is increasingly data intensive and recent technological developments, computational abilities, and new digital environments are placing data into the center of scientific discovery [1]. This “Fourth Paradigm” of data-intensive scientific discovery is built on three pillars of “capture, curation, and analysis” [2]. With data becoming a centerpiece of modern scientific discovery, data sharing by scientists is now a crucial element of scientific progress.

Data sharing is also a foundation for Open Science, the initiative “to make scientific research and data accessible to all. It includes practices such as publishing open scientific research, campaigning for open access and generally making it easier to publish and communicate scientific knowledge… [including] ways to make science more transparent and accessible during the research process” [3]. On January 15, 2019, U.S. President D. Trump signed into law H.R. 4174, the Foundations for Evidence-Based Policymaking Act of 2018, which supported implementation of the principles of Open Science in the United States: “[the law] improves evidence-based policy through strengthening Federal agency evaluation capacity; furthering interagency data sharing and open data efforts; and improving access to data for statistical purposes while protecting confidential information [4].”

The goals of Open Science include greater interdisciplinary scientific collaboration, accessibility of data, and greater reproducibility and transparency of scientific work. These are dependent on increased sharing of scientific data and open access data. Data sharing is increasingly seen as an essential driver of the direction in which science is moving worldwide and across disciplines [5, 6, 7].

Sound data management practices are required to achieve the goals of Open Science. Best practices in data management require scientists to share their data by depositing datasets in trusted subject, governmental, or institutional repositories, by providing metadata that makes their data findable, and by citing or acknowledging their reuse of data. Understanding the actual behaviors of scientists is key to understanding what can be done to support the scientific community using the best practices.

**Fig 1:**
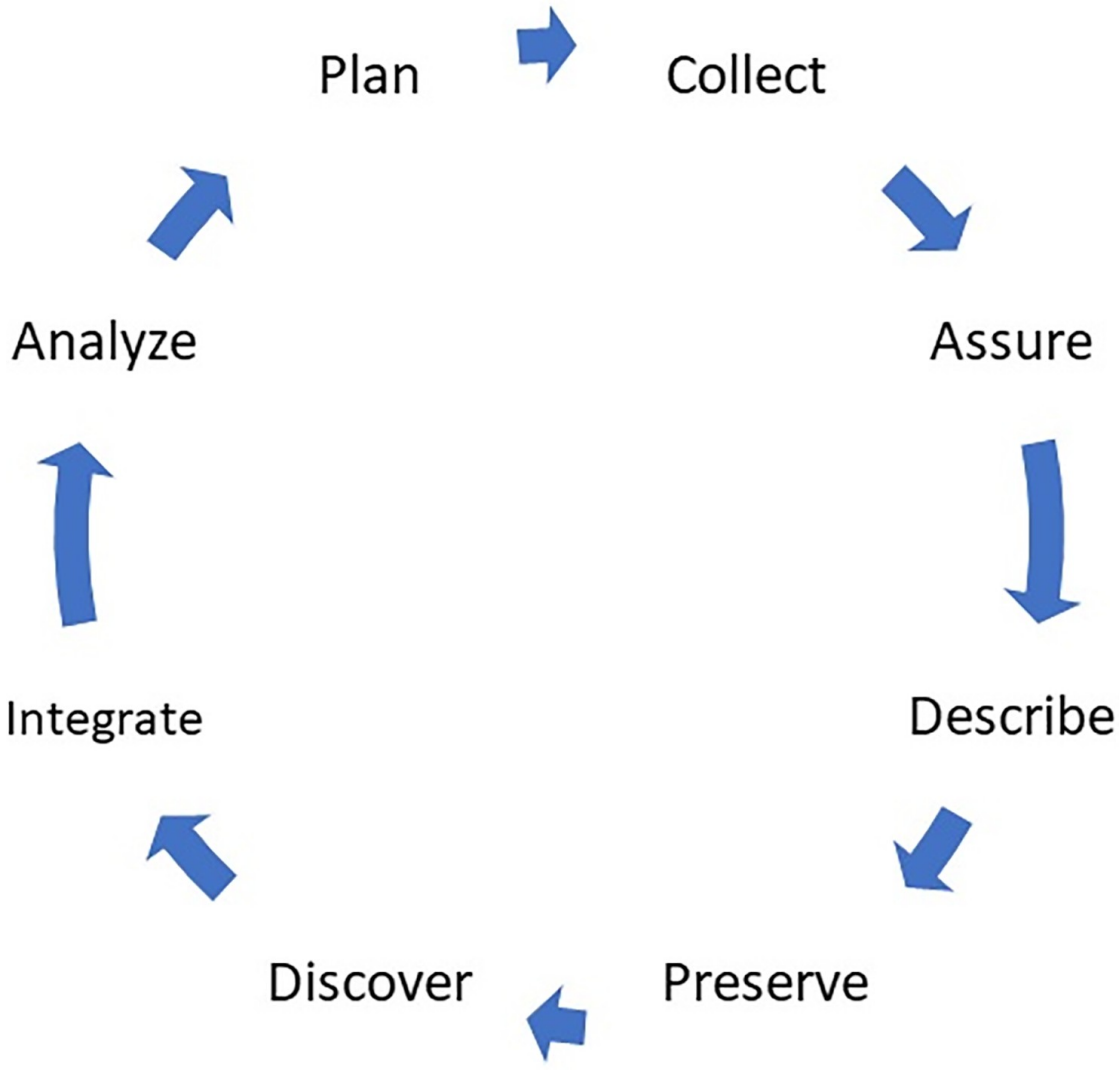
Data life cycle (from https://www.dataone.org/data-life-cycle).

Data management best practices are required throughout the full data lifecycle (Fig 1) and are well described in the “FAIR Guiding Principles for scientific data management and stewardship [8, 9]” which outlines a set of guiding principles to make data Findable, Accessible, Interoperable and Reusable. FAIR provide “guidance for scientific data management and stewardship and are relevant to all stakeholders in the current digital ecosystem. They directly address data producers and data publishers to promote maximum use of research data [10].” FAIR has been supported by many communities including the G20 Summit in 2016 and the Association of European Research Libraries (LIBER) in 2017 [10, 11, 12, 13]. A coalition of groups representing the Earth and space sciences headed by the American Geophysical Union (AGU) in 2017 set out to develop standards that will connect researchers, publishers, and data repositories in the Earth and space sciences to enable FAIR principles [14].

In 2018, LIBER published an Open Science Roadmap that outlined the specific actions that libraries could take to promote the concept and implementation of open science [11].

Other U.S. and international organizations, societies, and projects have also endorsed and are actively moving toward supporting these concepts and principles.

DataONE is an environmental cyberinfrastructure project focused on meeting “the needs of science and society for open, persistent, robust, and secure access to well-described and easily discovered Earth observational data… [created to] ensure the preservation, access, use and reuse of multi-scale, multi-discipline, and multi-national science data via three primary cyberinfrastructure elements and a broad education and outreach program.” DataONE has been supported by the U.S. National Science Foundation since 2009. DataONE Usability & Assessment Working Group (UAWG) has been studying scientists’ attitudes towards and practices with data sharing and reuse for a decade [5, 6]. Our paper reports on the results of our third survey conducted with scientists from a variety of subject disciplines and in many different countries.

Our study explores scientists’ data sharing attitudes and practices by focusing on specific stages of the data lifecycle including describe, preserve, and discover. It also examines where scientists store data both in the short and long-term, what metadata standards scientists use (if any) to describe data, and what barriers and incentives scientists face when data sharing, finding data and reusing data.

An analysis of the subset of this data, focused particularly on practices and attitudes of geophysicists and distributed to the members of the American Geophysical Union (1,372 responses from 116 countries) was published last year [15].

### National and institutional factors impacting data sharing

In the past decade, governments, funding agencies, and publishers have begun implementing more rigorous open access data policies and mandates [16, 17]. In the United States, the White House Office of Science and Technology published a memorandum in 2013 directing all federal agencies to increase public access to research and specifically allow open access to data and scholarly publications supported by federal funding [18]. A number of major U.S. federal funders have increased access to the results of funded research [8]) through a variety of policies including requiring funded projects to share data underlying the research when the results of the research are published. Some U.S. funding agencies, including the Centers for Disease Control (CDC), the Department of Defense (DOD), the Food and Drug Administration (FDA), the National Aeronautics and Space Administration (NASA), and the United States Agency for International Development (USAID), require grant recipients to submit a yearly data sharing plan as part of the application process.

The National Science Foundation [19] requires applicants to submit a data management plan as a part of the funding application, and encourages data sharing. “Investigators are expected to share with other researchers, at no more than incremental cost and within a reasonable time, the primary data, samples, physical collections and other supporting materials created or gathered in the course of work under NSF grants. Grantees are expected to encourage and facilitate such sharing [19].” The U.S. Geological Survey (USGS) Public Access Plan “outlines a framework for activities to increase public access to scholarly publications and digital scientific data resulting from research funded by the USGS [20]” and requires providing free public access to scientific data and information products that are developed or funded by USGS.

Over the past decade, the European Union and its member states have been implementing comprehensive open data policies. The European Strategy Forum on Research Infrastructures in 2010 stated that “data in their various forms (from raw data to scientific publications) will need to be stored, maintained, and made available and openly accessible to all scientific communities [1].” In 2012, the European Commission published a recommendation and a roadmap encouraging all member-states to make the publicly-funded research data and results accessible to the public [21]. Most public sector data in Europe is open, meaning it is widely accessible and available for reuse, sometimes with no restrictive conditions [22]. In addition, the EU has invested in digital public data infrastructure, launching two major online portals: the Open Data Europe Portal (ODP open-data.europa.eu/fr/data) “that harvests metadata from public sector portals throughout Europe… [and] focuses on data made available by European countries” and the European Data Portal (EDP europeandataportal.eu) which contains datasets collected and published by the European Institutions [23]. The European Commission in 2018 published a report and an action plan focused on implementing FAIR and providing recommendations and actions for stakeholders in Europe and beyond [23].

While requirements and regulations for open access to data vary regionally, data sharing practices of researchers worldwide are also affected by policies and regulations implemented by major stakeholders, such as publishers, journals and repositories. In addition to data sharing mandates by governments and funding agencies, changing professional codes of ethics and requirements by journals and publishers toward open data are resulting in broader support from research communities towards the practice [24]. Private foundations and other major funding organizations that require data sharing by researchers include, among others, the Bill and Melinda Gates Foundation, American Heart Association, and Howard Hughes Institute [25].

Many journals and societies (e.g., American Geophysical Union) require the deposit in appropriate public repositories of all data used in the studies reported in their publications [25, 26]. The National Academy of Sciences and its affiliates recommend that publishers implement open data access policies, and require researchers who want to be published to share their data [27].

### Individual factors impacting data sharing

Individual attitudes and practices related to data sharing may be at odds with journal or government mandates. The attitudes and practices of scientists have been investigated in a number of international and regional studies, which focus on current practices, willingness to share data, possible incentives to share, and perceived barriers [5, 6, 7, 28, 29, 30, 31].

In the past decade, these studies have shown that scientists increasingly recognize the real benefits of open data [32]. Our 2011 study found that a majority of the scientists were willing to share their data “if certain conditions are met (such as formal citation and sharing reprints) [5].” An expanded follow-up survey conducted several years later demonstrated “increased acceptance of and willingness to engage in data sharing, as well as an increase in actual data sharing behaviors [6].”

A 2016 international survey of scientists involved in environmental research found that 82% of respondents agreed that in their scientific community, open data was “very important,” and 17% thought it was of “intermediate importan(ce)” [7]. According to the same survey, support for data sharing “arose from research-intrinsic motives ranging from general considerations, i.e., the acceleration of scientific research and applications, to personal motivations, i.e., dissemination and recognition of research results, personal commitment to open data and requests from data users [7].” A study by Kim and Stanton [30] confirmed that individual factors that influence data-sharing behavior among scientists include perceived career benefit and career risk, scholarly altruism and the perceived amount of effort. Another study found that scientists seem to be more willing to share their data as a direct response to a request made by their peers, as they perceive this helps to ensure that their data will be cited and used properly [32].

Even though many funding agencies require data sharing, studies have shown that many scientists do not share their data, even those who receive funding from agencies that require data sharing [31, 6]. We found major barriers to sharing data are related to insufficient funding and time constraints to prepare the data for access and reuse [5] and these barriers are persistent and include concerns about the need to publishing their analysis first [6]. The Belmont Forum open data survey of environmental scientists produced similar results; the top barriers to publish data included the need to publish first, legal constraints and concerns about loss of credit of recognition [7]. According to the same survey, the age of the respondents had an impact on perceived barriers; “desire to publish results before releasing data was somewhat more prevalent at early stages of a research career [7].”

Scientists often explain their reluctance to share data as a concern that their data will be misused or misinterpreted [28, 7]. In disciplines that deal with human subjects, patients’ or respondents’ privacy concerns, as well as legal regulations, could be additional barriers to data sharing [28].

Another significant barrier, according to Gorgolewski, Margulies, and Milham [33] is researchers’ fears that reuse of their data and the resulting scrutiny by other researchers may reveal errors or discrepancies in the datasets, or in their interpretation.

Factors that impact researchers’ willingness to share data include 1) the extent to which they are trained in data management best practices, 2) the availability of organizational support to assist them, and 3) the extent to which they feel assured that the original datasets will be acknowledged and cited when reused [29]. Although there may be mandates to share data, the lack of perceived incentives for researchers to spend additional time and effort to prepare datasets for sharing is another reason why more data is not available publicly [28]. An opportunity to be a co-author on a study that uses their data could be a significant incentive for data sharing because of the need to publish scholarly articles for career advancement in academia [34]. Results of the Tenopir et al. [5, 6] surveys indicated significant disciplinary differences in perceived incentives and barriers, as well as the age of the respondents, as factors in data sharing attitudes and practices. While younger respondents generally expressed more positive attitudes towards data sharing, their responses demonstrated that in reality, they are sharing less of their data than older researchers.

In addition, the type of data used by scientists and the ease with which this data can be reused is affecting data sharing and reuse practices: “reported use of models and remote-sensed data had a large positive effect on reuse behavior [35]”. Kim et al. confirmed that types of “data sources used by academic researchers were found to have a significant relationship with data sharing and reuse behaviors [36]”.

While most researchers seem to be satisfied with the initial steps of the data lifecycle (searching for, collecting and short-term storage of data), long-term data preservation is much more challenging and problematic, and most organizations do not provide sufficient training and support for data management [5, 6]. Institutional involvement in training and data management assistance is crucial since the lack of skills and knowledge needed to prepare datasets for sharing is one primary reason why scientists choose not to share their data [31, 36].

In this study, we used surveys to examine attitudes and practices at three stages of the data lifecycle: description, preservation and discovery. In particular, our questions focused on where data are stored both in the short and long-term, what metadata standards are used (if any) to describe their data, and barriers and incentives to data sharing and discovery.

We sought to answer the following research questions:

- Where are the scientists storing their research data?
- Are the scientists satisfied with the process of storing their data during and beyond the life of research projects?
- How many scientists share data and how much of their data do scientists share?
- What are the attitudes toward sharing and reusing research data?
- Do scientists use sound data management practices, such as creating data management plans, providing metadata, and preserving data for the long term?
- What are the barriers and incentives for sharing research data?
- How much support and training in data management do organizations offer?
- How do attitudes and practices of data sharing differ by sub-discipline of science and primary work sector?

For the purposes of analysis in this paper we defined *good data practices* as those that support the FAIR data principles and are likely to guard against data loss, helps facilitate sharing with other researchers, and helps facilitate long-term curation. Good data practices were assigned when respondents indicate they store data in a repository. In contrast, *mediocre data practices* are operationally defined as practices that have the potential of making data findable with some additional effort, but do not obviously support FAIR principles, such as storing data in a personal cloud or on an institutional/departmental/PI’s server. Finally, *bad data practices* are defined as those that put data at risk and make it difficult to find or preserve, such as storing data on a flash drive, personal computer, or on paper.

## Material and methods

We conducted a survey distributed in a two-waves approach with the assistance of the American Geophysical Union (AGU) and several partners and colleagues. The first wave of the survey was conducted in 2017–2018, when AGU distributed an email with a link to the instrument to all 62,000 of its members. An email reminder was sent out in August 2017, and the survey was closed on March 2018 with 1372 responses for a response rate of approximately 2.2%.

The second wave was distributed between late 2017 to Spring 2018 by a variety of organizations, including the Ecological Society of America, United States Geological Survey, Elsevier, Wiley, Baltic Association of Media Research, and LabArchives. The survey was also distributed by a number of colleagues in the Middle East, Eastern / Central Europe, and Eastern and Northern Europe. The survey link was disseminated both through email and by posting a link to the survey on Twitter. The second wave of the survey ended on May 11, 2018, with 812 responses. Combined, the two waves of the survey include 2184 responses. Survey data were collected in Qualtrics and housed on a secure server at the University of Tennessee. Researchers used IBM SPSS 25 statistical analysis software package for data analysis.

The study was approved by the University of Tennessee Institutional Review Board (IRB) as an online survey that did not gather personally identifiable information. Findings are reported in aggregate and do not contain any personally identifiable information. The informed consent agreement asked respondents to indicate that they understood the terms and were over 18 years of age. In compliance with the IRB approval for work with human subjects, respondents could skip any question or withdraw from the study at any time.

### Research instrument

This survey was developed based on two previous surveys of scientists conducted by members of the DataONE Usability and Assessment working group [5, 6] For consistency, questions from previous surveys were reviewed and most previous questions were kept in the new survey. Some new questions and potential responses were added to reflect recent changes and developments in data management. Some questions that were less relevant were removed. The survey consisted of two parts, including the demographics section and a section exploring data use and reuse, data storage, and data sharing.

In the section focused on demographics, respondents were asked about their primary sector of employment, primary subject discipline, type of research activity, primary place of employment, as well as the year of birth, highest degree attained, and their gender. We decided to use chronological age instead of career stage to be consistent with our previous studies to see whether age has an influence on data attitudes or practices.

In order to explore a respondent’s practices and attitudes towards data sharing, use, and reuse, the survey included multiple questions focused on each of those topics. The survey consisted of questions asking participants to express the degree to which they agree or disagree with various statements, as well as several yes/no questions.

To discover the attitudes and practices of scientists regarding data, respondents were asked questions focused on various aspects of data management, including their current data practices, available support, practices of organizations and institutions that fund or employ them, data reuse, sharing and barriers, metadata, and institutional frameworks. In the event that the selection of possible responses did not accurately represent the respondents’ opinion, an option to select "Other" and write-in response was included in several questions.

## Results

**Fig 2:**
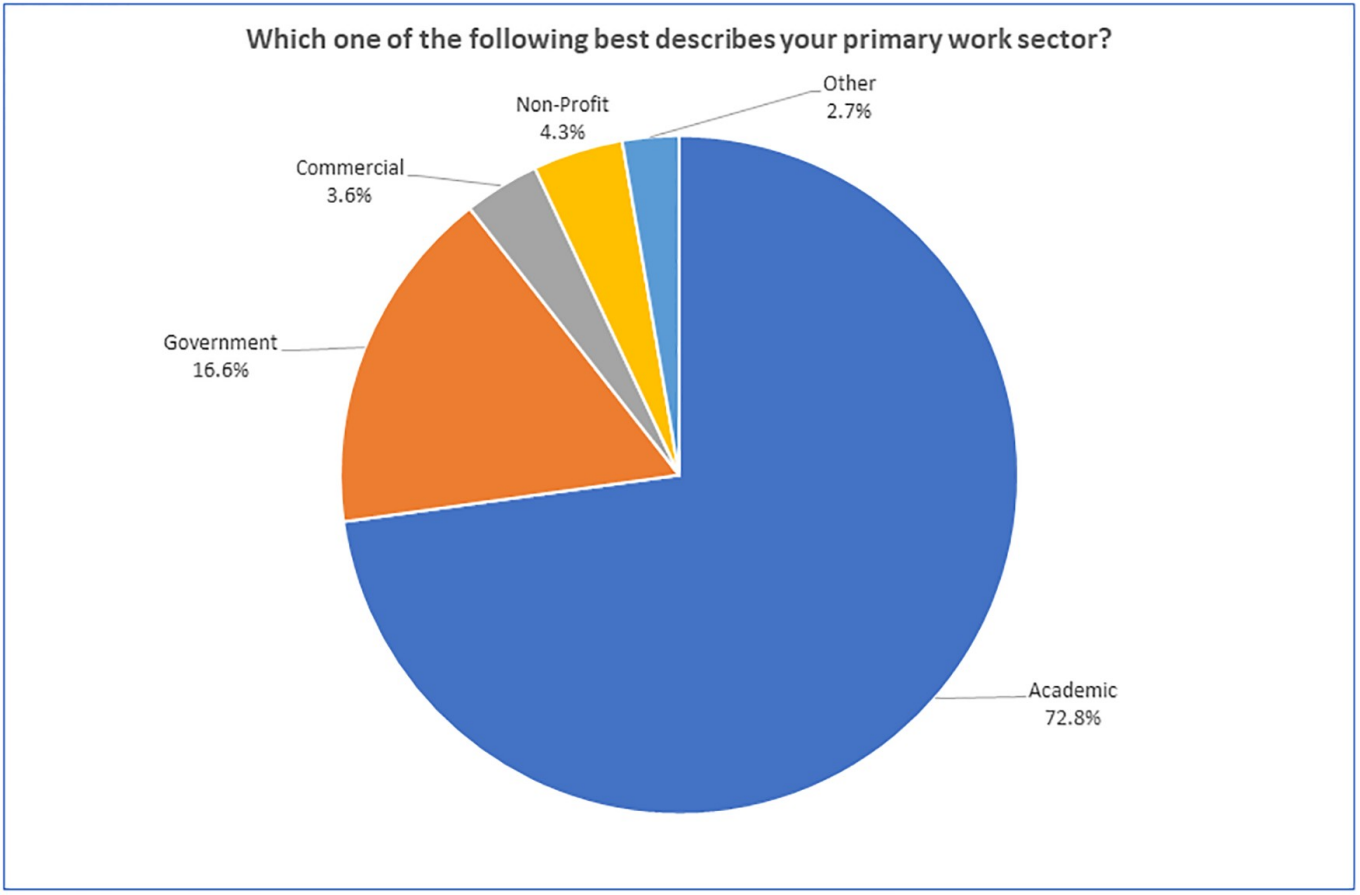
“Which one of the following best describes your primary work sector?” (n = 2088).

Almost two-thirds of the respondents who answered the question regarding their primary work sector (see Fig 2) were employed in the academic sector (72.8%), followed by government (16.6%), commercial (3.6%), non-profit (4.3%) and other (2.7%).

Our analysis combines the described disciplines into four categories. The largest group of respondents are in the physical sciences (43.3%), a quarter are in life sciences (26.3%), and 10.7% say their discipline was computer science and engineering (see Table 1). The rest of the respondents (19.8%) represent various other disciplines. For the full list of disciplines, see S1 Table.

**Table 1:** “Which one of the following best describes your primary subject discipline?” (grouped) (n = 2098).

Discipline (grouped)	%
Physical Sciences (atmospheric/hydrology/physical/geology)	43.3%
Life Sciences (bio/ecology/environmental/marine/agriculture)	26.3%
Other (other/social science)	19.8%
Computer Science and Engineering	10.7%

**Fig 3:**
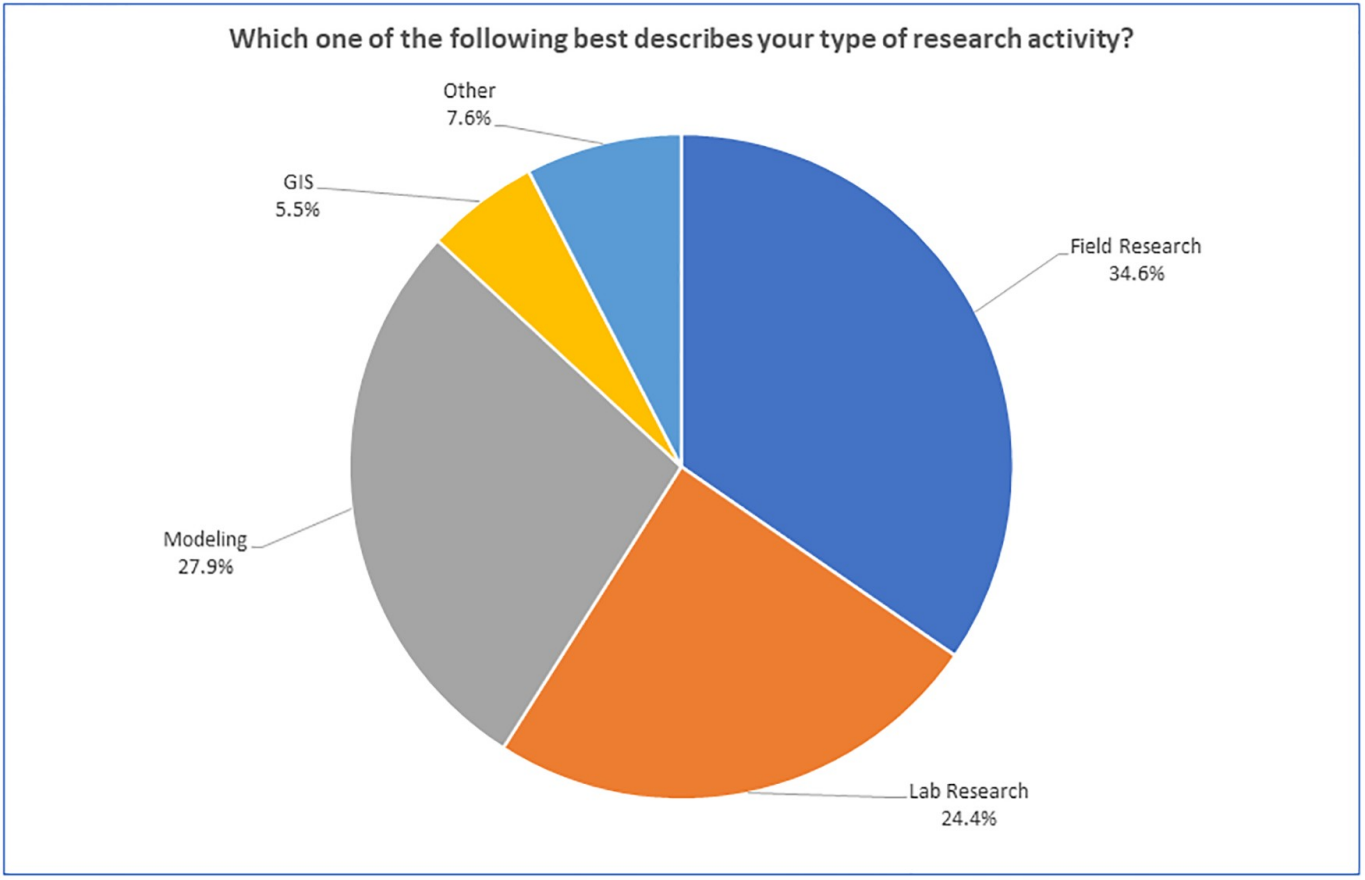
“Which of the following best describes your primary type of research activity?” (n = 2032).

Over a third of respondents said they primarily conduct field research (34.6%); another third primarily engage in modeling (27.9%); and a quarter conduct lab research (24.4%) (see Fig 3).

**Fig 4:**
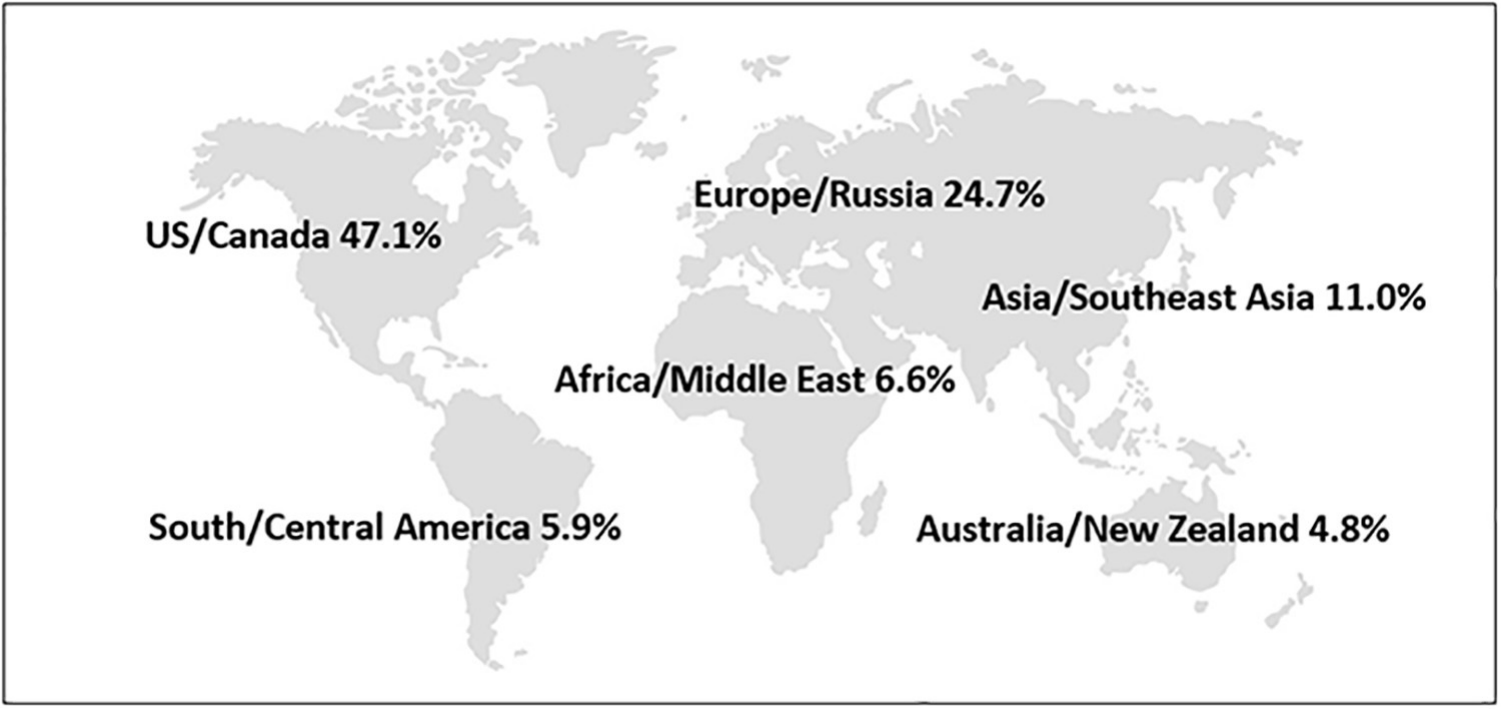
“Primary place of employment” grouped by region (n = 2016).

Respondents identified 87 different countries when asked about the location of their primary place of employment with 44.6% of respondents indicating their primary employment was in the United States. Only eight countries had over 50 respondents. Our analysis groups countries of employment into six regions. Almost half of the respondents were employed in U.S./Canada (47.1%), followed by Europe/Russia (24.7%); Asia/Southeast Asia (11.0%); Africa/Middle East (6.6%), South and Central America (5.9%), and Australia/New Zealand (4.8%) (see Fig 4). Analysis of the regional differences of this survey will be published in a forthcoming manuscript.

The original year of birth variable was transformed into age and our analysis groups these into five different clusters. Of the respondents who answered this question (n = 1984), 15.2% are 29 years old and under, 28% are 30–39 years old; 21.8% are 40–49 years old; 18.6% are 50–59 years old; and 16.3% are over 60.

Among those respondents who answered the gender question (n = 2003), 65.5% specified male, 32.2% specified female, and 2.3% preferred not to answer.

Of those respondents who answered the question “Does your primary funding agency require you to provide a data management plan?" (n = 1966), almost half (48.6%) said yes, just over a third said no (38.2%), and 13.2% did not know if their primary funding agency required a data management plan.

### Current data practices

#### Data use

Using best practices when storing data is important both during the life of the project and beyond the life of the project. Short-term storage was defined as “storing my data during the life of the project” while long-term was “storing my data beyond the life of the project.” Typically, data storage practices vary and may reflect convenience rather than consideration of sound storage practices.

Responses to the question “How much of your data do you currently store or deposit in the following locations?” indicated that a personal computer was the primary location for data storage (61.3% of respondents store all or most of their data on a personal computer), followed by the respondent’s institution’s server (42.9%) and USB/external drive (29.8%). This question was not specifically focused on short-term or long-term data storage practices, but was referring to general data storage practices. Safer storage options such as cloud storage or repositories of various kinds are used far less often for data storage (Table 2).

**Table 2:** “How much of your data do you currently store or deposit in the following locations?”. (This is a combined number of the respondents who store most or all of the data in the specified location. Each response option was coded as a separate question, since the respondents may store data in various locations simultaneously, and answer “Not sure” is omitted from the table).

Storage location	None	Some	Most	All	n*
On my institution’s server	469 **(27.2%)**	468 (27.1%)	333 (19.3%)	407 (23.6%)	1727
On the principal investigator’s server	655 **(41.0%)**	342 (21.4%)	240 (15.0%)	282 (17.6%)	1598
On a departmental server	809 **(52.0%)**	342 (22.0%)	192 (12.3%)	146 (9.4%)	1556
On my personal computer	161 (9.2%)	503 (28.7%)	356 (20.3%)	718 **(41.0%)**	1753
On paper in my office	698 **(44.4%)**	654 (41.6%)	116 (7.4%)	81 (5.1%)	1573
USB/external drive	557 **(35.6%)**	496 (31.7%)	184 (11.8%)	281 (18.0%)	1565
In a discipline-based repository, (e.g. NEON or LTER)	1074 **(49.2%)**	218 (10.0%)	92 (4.2%)	31 (1.4%)	1536
In a publisher or publisher-related repository (e.g., specific publisher or Dryad)	1030 **(66.8%)**	334 (21.7%)	53 (3.4%)	19 (1.2%)	1541
Other data repository or archive (e.g., national data center)	850 **(53.7%)**	383 (24.2%)	192 (12.1%)	72 (4.5%)	1584
In my institution’s repository	858 **(55.2%)**	357 (23.0%)	133 (8.6%)	122 (7.9%)	1553
Cloud storage	686 **(43.1%)**	479 (30.1%)	184 (11.6%)	191 (12.0%)	1593
Other	713 **(72.8%)**	56 (5.7%)	18 (1.8%)	33 (3.4%)	979

**Fig 5:**
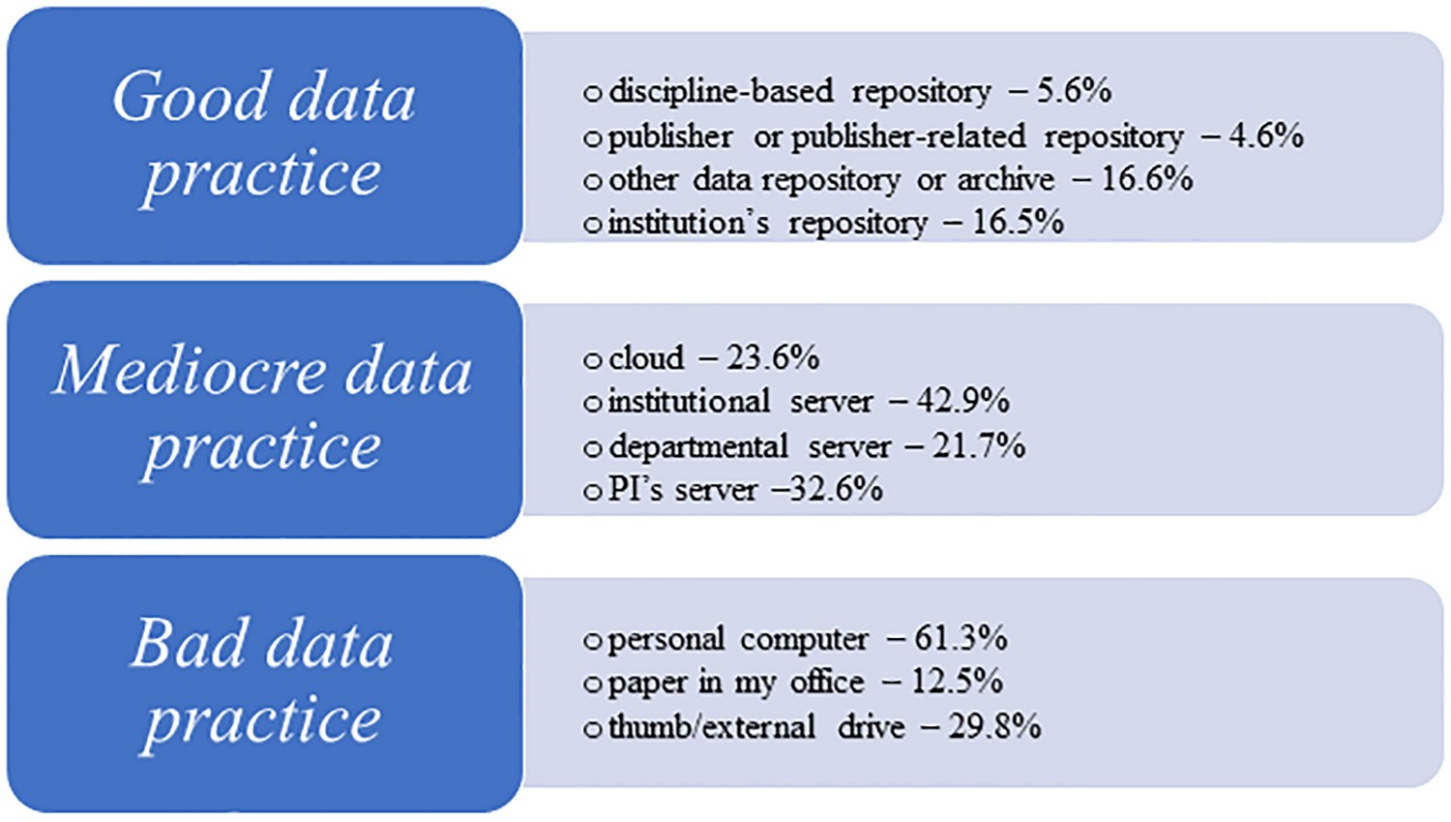
How much of your data do you currently store or deposit in the following locations? (The number is a sum of answers “Most data” and “All data.”).

To better differentiate between what are considered better data storage practices than others, we recombined various data storage and deposit practices into three groups. We defined *good data practices* as storing data in a repository of some kind; *mediocre data practices* as storing data in the personal cloud, or on an institutional/departmental/PI’s server; and *bad data practices* as storing data on a flash drive, personal computer, or on paper. Fig 5 represents the number of responses for each data storage group and clearly shows that mediocre or bad data storage practices are much more common. Good data practices were the least popular option, with over half of the respondents indicating that they did not store any of their data in any kind of repository.

Data storage practices varies greatly across age groups (Chi-Square = 21,063; p = .000): the younger the age of the respondents, the less likely they were to adhere to good data practices. While over a third of respondents who were fifty years or older demonstrated good data practices, this number was lower in each of the younger age groups; in the age group of 40–49 year old it was under a quarter of respondents, and among those who were 30–39 years old, it was 21.6%. In the youngest age group (under 29 years old) only 19.2% of respondents demonstrated good data storage practices.

There are also disciplinary differences (Pearson Chi-Square = 52,949; p = .000) in stated data storage practices. In Marine Sciences nearly half (46.5%) of respondents report good data storage practices, followed by Space and Planetary Science (36.4%), Atmospheric Science (31%) and Environmental Science (28.3%). In the rest of the disciplines, the number of scientists who report good data storage practices is between a quarter and a fifth of the respondents. Biology was the last discipline on the list, with only 11.4% of biologists adhering to good data storage practices. Analysis by the sector of employment suggest that governmental (33.5%) and non-profit employees (28.1%) are leaders in good data storage practices, followed by the commercial sector (23.7%), academia (21.9%), and other sectors (14.3%).

**Fig 6:**
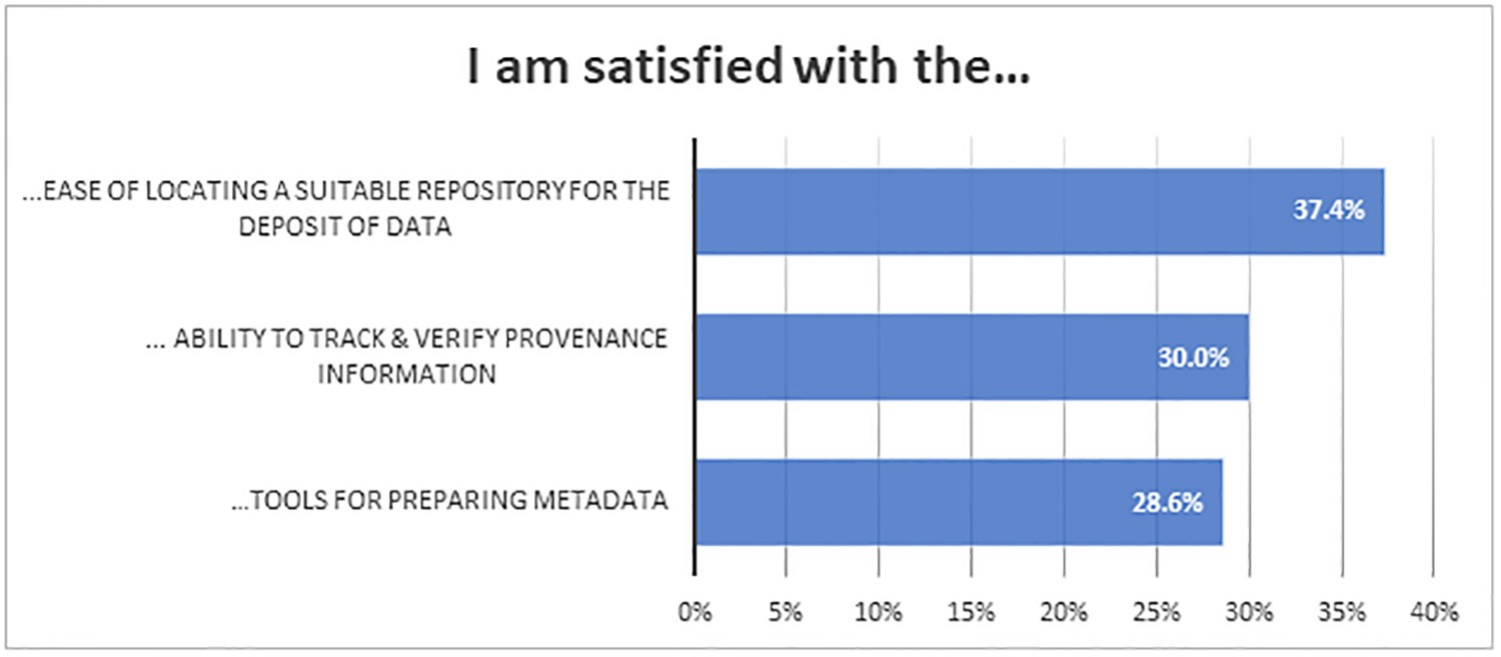
“I am satisfied with the …”. (Locating suitable repository n = 1812; provenance information n = 1803; tools for preparing metadata n = 1808).

Even if researchers are not using good data storage practices, researchers seem satisfied with their own practices (Fig 6). This mismatch between good practices and satisfaction may show that data storage is less important to them than data collection and analysis. Approximately three-quarters of respondents (74.5%) report they are satisfied with the process of storing their data during their project (short-term). However only half (52.4%) are satisfied with the process of storing data beyond the life of the project (long term).

Chi-square tests showed that satisfaction with short-term data storage had some disciplinary variation (Pearson Chi-Square = 72,280; p = 0.01). Disciplines where almost three-quarters of respondents expressed satisfaction with short-term storage included Marine/Ocean (79.1%), Psychology (77.3%), and Biology (76.3%). The lowest level of satisfaction was from respondents representing Other Sciences (58.7%), Information Science/Computer Science (59.6%) and Physical Science (59.8%).

Satisfaction with data storage beyond the life of a project also has disciplinary variation. Respondents with the highest level of satisfaction with long-term data storage were Marine/Ocean (55.8%), Biology (50.9%) and Agriculture and Natural Resources (52.1%). The least satisfied were respondents from Space and Planetary Science: less than a third of them (27.3%) said they were satisfied with the process of storing data beyond the life of a project.

Attitudes towards both short-term (Pearson Chi-Square = 68.926; p = .002) and long-term (Pearson Chi-Square = 52.175; p = .000) data storage show variation across the age groups. The older the age of participants, the more satisfaction they expressed both with short-term data storage and long-term (Table 3).

**Table 3:** “The following statements relate to how you store and manage your data. Tell us how much you agree with the following ways to complete the sentence: I am satisfied with…”.

Answer	60+	50–59	40–49	30–39	Under 29
… process of storing my data during the life of the project (short-term)	244 (75.3%)	274 (74.1%)	284 (65.7%)	354 (63.7%)	185 (61.3%)
… process of storing my data beyond the life of the project (long-term)	183 (56.5%)	201 (54.3%)	203 (47.0%)	236 (42.4%)	120 (39.7%)

When researchers are seeking data to answer their research questions, over three-quarters rely on themselves or their colleagues and about two-thirds of respondents state that they search for existing data. However, most researchers are making this search without consulting a data manager or librarian.

The fact that the respondents consult with a data manager or librarian may reflect the availability of those resources. While the age of the respondents did not have a significant impact on their reaching out to librarian or data manager for help acquiring the data, a Chi-square test (Pearson Chi-Square = 12.283; p = .015) showed a statistically significant relationship between the sector of employment and the respondent’s habit of asking data managers for help. The highest prevalence of those who ask data managers for assistance is in the commercial sector (33.4%), followed by non-profit (21.5%) and the government (20.0%). The lowest percentage of respondents who reach out to data managers (only 17.3%) are employed in the academic sector.

There are statistically significant variations by discipline in regards to working with both librarians (Pearson Chi-Square = 92.647; p = .000) and data managers in seeking new data (Pearson Chi-Square = 30.667; p = .004). Respondents from the following disciplines were more accustomed to consulting with librarians: Information/computer science (35.8%) Engineering (33.6%); other (34.0%) and Agriculture/Natural Recourses (27.6%). In all other disciplines, less than a fifth of respondents asked librarians for assistance with data.

A similar disciplinary variation is evident in the willingness of the scientists to ask data managers for help: respondents representing Agriculture and Natural Recourses (29.7%), Information Science (28.1%) and Engineering (25.9%) are most likely to consult with data managers. In all other disciplines, just 10 to 20 percent of respondents indicated they consult with data managers.

#### Data management support and practices

The results of the survey highlighted the need for organizations to offer more formal training and assistance in data management to scientists, or to better publicize the support they do offer. Overall, only about a third of the respondents stated that their organizations provide any training or assistance.

When asked what kind of assistance or training is provided by the respondent’s organization or project, about a quarter to a third of respondents said they are provided with assistance in creating data management plans (33.3%); training on best practices in data management (31.3%); assistance on creating metadata to describe data or datasets (27.6%); and training on data citation (27.6%). There were differences amongst work sectors in both the types and extent of training and assistance in data management provided by organizations (Table 4). Government sector respondents indicated the highest rates of provided training and assistance, while the employees of the academic sector reported the lowest rates. With respect to training on best practices, assistance creating data management plans, and help developing metadata, the correlation to work sector was statistically significant. While there was some variation among different sectors on training for data citation, it was not statistically significant.

**Table 4:** Crosstab between “Primary sector of employment” and “My organization or project provides (training/assistance)…”.

Assistance/training	Academic	Government	Commercial	Non-profit
…training on best practices for data management (Chi-Square = 31.923; p = .000)	374 (28.4%)	127 (42.2%)	23 (39.0%)	24 (34.8%)
…assistance on creating data management plans (Chi-Square = 49.543a; p = .000)	384 (29.2%)	140 (46.8%)	25 (43.1%)	31 (45.6%)
…assistance on creating metadata to describe my data or datasets. (Chi-Square = 102.006; p = .000)	288 (22.1%)	147 (49.0%)	20 (33.9%)	26 (38.2%)
…training on how to cite datasets. (Chi-Square = 10.421a; p = .200	329 (25.1%)	93 (31.1%)	14 (23.7%)	19 (27.9%)

There were noteworthy disciplinary differences regarding training and assistance in data management provided by the organization (see Table 5).

**Table 5:** Crosstab between “Primary Subject Discipline” and “My organization or project provides …”.

	…training on best practices for data management (Chi-Square = 68.580; p = .000)	…assistance on creating data management plans (Chi-Square = 62.312; p = .000)	…assistance on creating metadata to describe my data or datasets (Chi-Square = 74.476; p = .000)	…training on how to cite datasets. (Chi-Square = 44.938; p = .012)
Agriculture and Natural Recourses	35 (42.7%)	30 (37.0%)	24 (29.3%)	23 (28.4%)
Atmospheric science	51 (26.6%)	66 (33.2%)	68 (34.2%)	47 (23.5%)
Biology	37 (34.6%)	38 (35.5%)	24 (22.4%)	21 (19.6%)
Information/Computer science	32 (47.8%)	34 (50.0%)	28 (41.2%)	25 (37.3%)
Environmental Science/Ecology	93 (33.0%)	106 (37.7%)	92 (32.7%)	72 (25.6%)
Engineering	33 (26.8%)	33 (27.0%)	32 (26.2%)	35 (28.2%
Geology/Earth Science	76 (24.1%)	82 (26.4%)	64 (20.6%)	68 (21.9%)
Hydrology	31 (34.8%)	32 (36.0%)	31 (34.8%)	28 (31.5%)
Physical sciences	57 (29.4%)	61 (31.3%)	53 (27.3%)	51 (26.2%)
Psychology	7 (31.8%)	6 (28.7%)	2 (9.1%)	3 (13.6%)
Other	87 (34.9%)	84 (34.1%)	52 (21.6%)	74 (30.6%)
Marine/Ocean	18(46.2%)	18 (46.2%)	17 (43.6%)	14 (35.9%
Space and Planetary Science	2 (11.1%)	4 (22.2%)	4 (22.2%)	4 (22.2%)

Researchers’ levels of satisfaction with tools and practices associated with data management were low: only about a third of the respondents expressed satisfaction with the data tools and practices listed in Fig 6.

There was some work sector variation in respondents’ satisfaction with tools and practices (Table 6). Government employees were most satisfied with tools for preparing metadata (29.8%), while respondents from the commercial sector were the least satisfied with those tools (17.1%). In all other sectors, approximately a quarter of respondents expressed satisfaction with the metadata tools.

**Table 6:** Crosstab between “Primary sector of employment” and “Satisfaction with tools for metadata, provenance information and repositories”.

	Academic	Government	Commercial	Non-Profit
… ability to track & verify provenance information (Chi-Square = 34.813; p = .001).	388 (25.5%)	101 (29.2%)	22 (28.9%)	18 (20.2%)
…ease of locating a suitable repository for the deposit of data (Chi-Square = 29.455; p = .003).	480 (31.6%)	135 (39.0%)	25 (32.9%)	22 (24.7%)

Levels of satisfaction with the ability to track and verify provenance information (Chi-Square = 24.456; p = .002), as well as the ease of locating a suitable repository for the deposit of data, also varied among work sectors. Here, again, governmental employees showed the highest level of satisfaction of all work sectors, while respondents from the non-profit sector expressed the least satisfaction.

Satisfaction with available metadata tools also varied significantly by the primary subject discipline of the respondents. Respondents who indicated that their primary discipline was Space and Planetary Science (36.4%), Atmospheric Science (32.3%), and Hydrology (27.3%) expressed a higher degree of satisfaction with the tools for preparing metadata, while those in Geology (21.8%), and Biology (15.8%) expressed the least satisfaction with the metadata tools (Chi-Square = 59.157; p = 000).

Respondents’ abilities to easily locate a suitable repository also differed across disciplines (Chi-Square = 62.686; p = 000). Respondents representing Space and Planetary Science (45.5%), Information/Computer Science (40.4%), and Geology/Earth Science (38.9%) stated that it was easy for them to locate a data repository, while for the scientists in Agriculture and Natural Resources (28.7%), Biology (27.2%) and Other Sciences (25.5%) it seemed to be a more laborious task.

#### Data sharing and reuse

**Fig 7:**
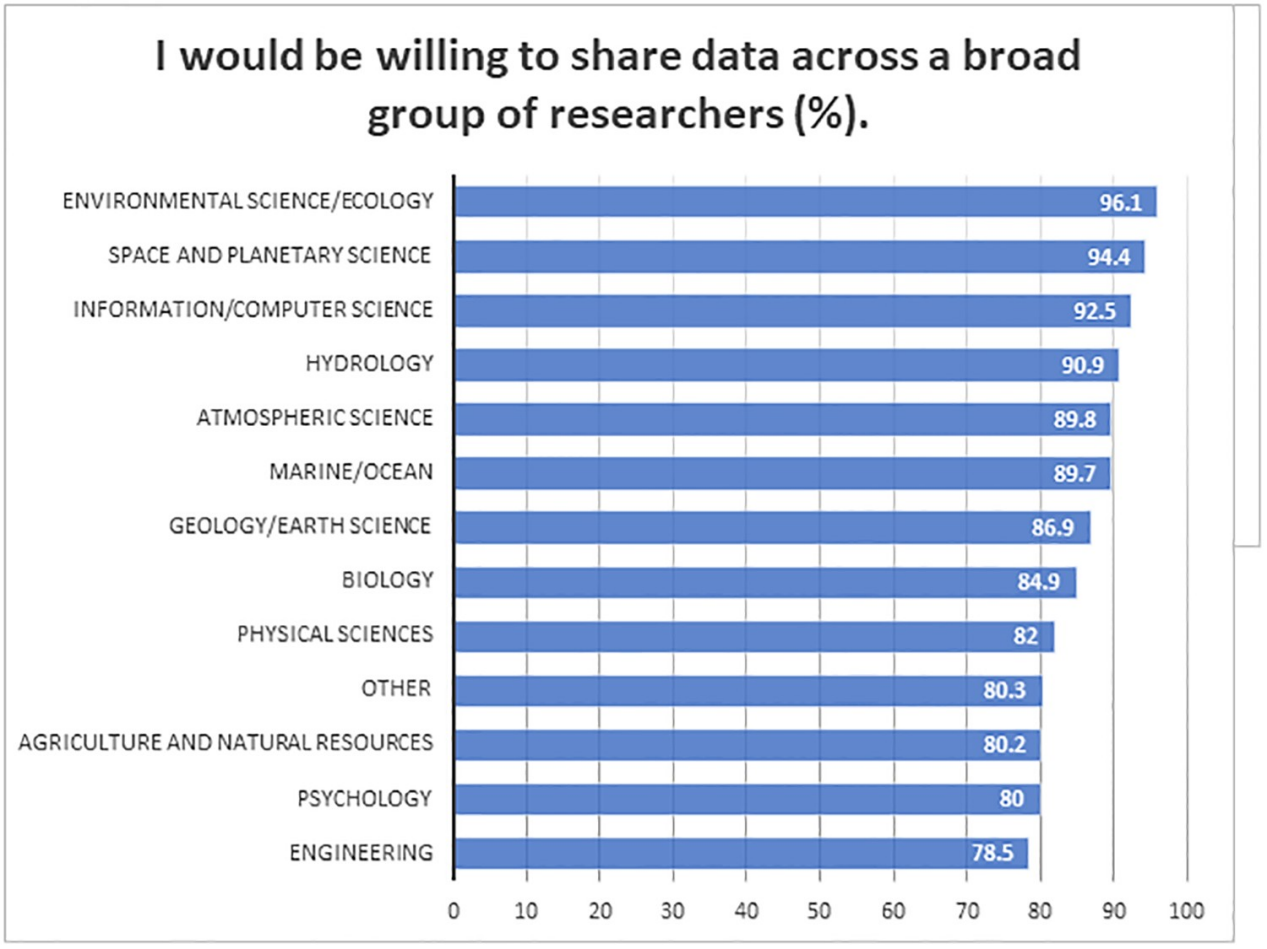
Cross between “primary subject discipline” and “I would be willing to share data across a broad group of researchers (n = 1776).

The idea of using data produced by other researchers was viewed positively by the vast majority of respondents: 87% said they would use such data if it would be easily available. Respondents were also enthusiastic about sharing their own data—86.7% said they are willing to share data across a broad group of researchers. There was only a slight variation between sectors of employment: respondents from the commercial sector were slightly less willing to share their data (72.1%), while in all other sectors the number of those willing to share ranged from 86.7% to 89.6%. Fig 7 shows the disciplinary variation in the willingness of respondents to share their data:

Respondents representing Environmental Science (96.1%), Space and Planetary Science (94.4%), Information/Computer Science (92.5%) had the most positive attitudes towards sharing their data. Three-quarters of the respondents (77.3%) said that they would be willing to place at least some of their data into a central data repository with no restrictions (Table 7). Respondents were more hesitant about the idea of sharing all of the data: less than half of respondents (44.5%) said they would be willing to share all of their data with no restrictions. Around half of respondents (56.4%) said that they would be more willing to share data if they could place some conditions on access. At the same time, for the vast majority of respondents (92.1%) getting cited by users of their datasets was important.

**Table 7:** “The following statements relate to sharing scientific data. Tell us how much you agree with each statement”. (Answers “Agree Somewhat” and “Agree Strongly” combined).

Answer	n (%)
I would use other researchers’ datasets if their datasets were easily accessible.	1794 (87.0%)
I would be willing to place at least some of my data into a central data repository with no restrictions.	1785 (77.3%)
I would be willing to place all of my data into a central data repository with no restrictions.	1778 (44.5%)
I would be more likely to make my data available if I could place conditions on access.	1775 (56.4%)
I am satisfied with my ability to integrate data from disparate sources to address research questions.	1774 (49.8%)
I would be willing to share data across a broad group of researchers.	1776 (86.7%)
It is important that my data are cited when used by other researchers	1777 (92.1%)
It is appropriate to create new datasets from shared data.	1776 (70.3%)

Lack of access to data generated by others was seen by most respondents (74.6%) as a major impediment to science, but only half (50.5%) thought it affects their own personal ability to answer scientific questions (Table 8).

**Table 8:** “The following statements relate to your views on the use of scientific research data. Tell us how much you agree with each statement”.

Answer	n (%)
Lack of access to data generated by other researchers or institutions is a major impediment to progress in science.	1777 (74.6%)
Lack of access to data generated by other researchers or institutions has restricted my ability to answer scientific questions.	1770 (50.5%)
Data may be misinterpreted due to complexity of the data.	1769 (78.7%)
Data may be misinterpreted due to poor quality of the data.	1764 (78.6%)
Data may be used in other ways than intended.	1765 (75.4%)

When asked what would increase their confidence in using data collected by others, the vast majority (82.1%) of respondents thought it most important to see written details about collection and quality assurance methods accompanying the data, followed by explicitly stated metadata standards (69.1%) and detailed information about the provenance (60.9%) (Table 9).

**Table 9:** “The following statements relate to your views on the reuse of scientific research data. Tell us how much you agree with the following ways to complete this sentence: I would have increased confidence in re-using data collected by others if…”.

Answer	n (%)
The metadata standard(s) utilized were explicitly stated with the data	1737 (69.1%)
The data were accompanied by written details about collection and quality assurance methods	1741 (82.1%)
A recorded workflow from a standard workflow system (Kepler, VisTrails, Taverna, etc.) was also available with the data	1738 (36.0%)
Detailed information about the provenance (data lineage, chain of custody) were available with data	1724 (60.9%)
Other	627 (21.9%)

**Fig 8:**
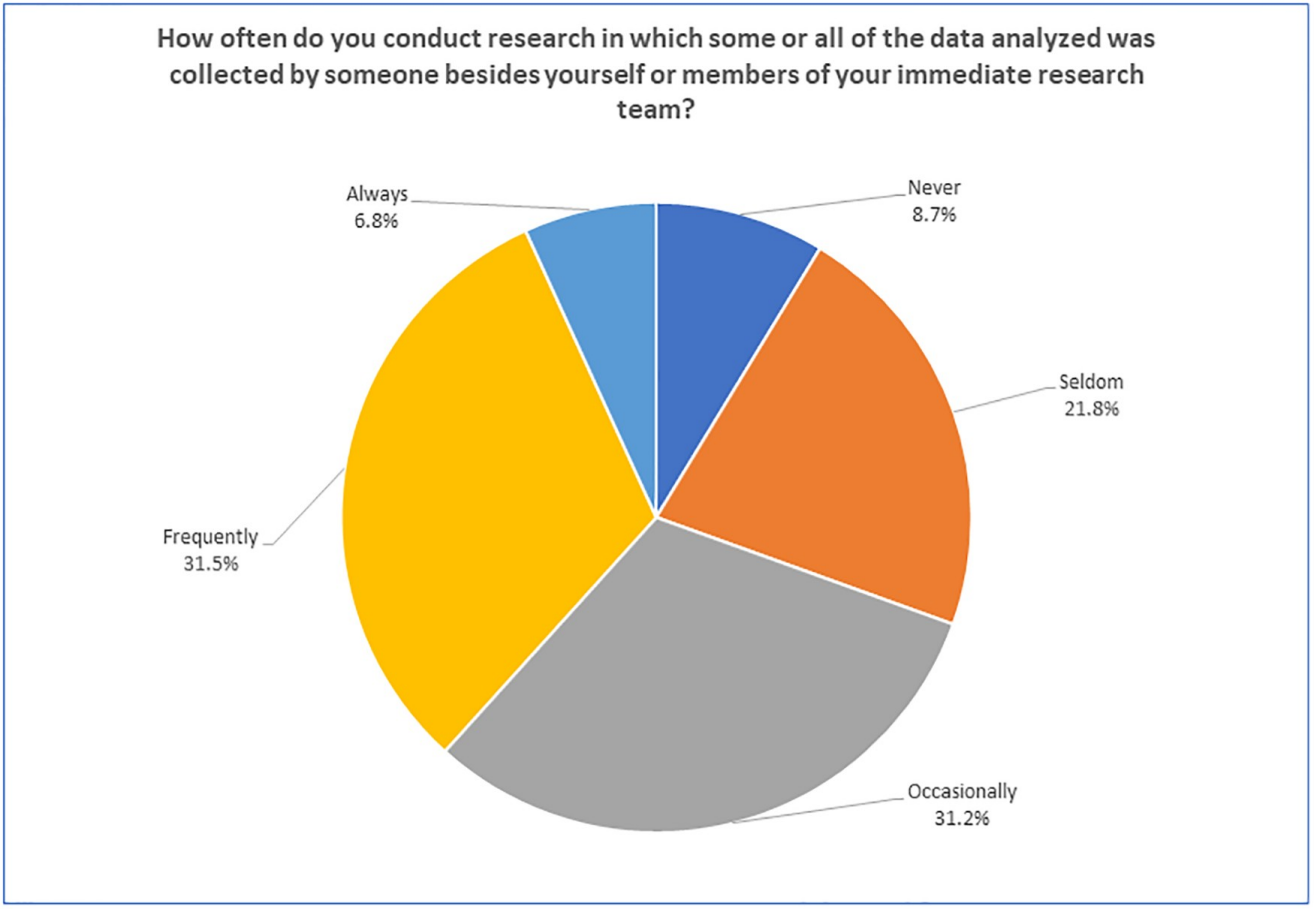
“How often do you conduct research in which some or all of the data analyzed was collected by someone besides yourself or members of your immediate research team?” (N = 1795).

Over a third of respondents (38.3%) report (see Fig 8) that they are regular users of data collected by others. We define “regular users” as those who answered “Always” or “Frequently” to the question about use of data collected by others.

There is, however, variation in the use of data collected by others, based on work sector and discipline. Government and commercial employees seemed to be using data collected by others more frequently; 59.1% and 50.1%, respectively, were regular users of such data. In the non-profit and academic sectors only about a third of respondents (36.2% and 34.8%) were regularly using data that they did not collect.

Responses to this question also varied by the primary subject discipline (Chi-Square = 87.461; p = .000). Respondents who indicated the highest rates (Always or Frequently) of regular use of data generated by others appear to be clustered in three disciplines: Atmospheric Science (50.4% of heavy users); Space and Planetary Science (45.5%), Marine/Ocean (46.5%); and Hydrology (44.0%). In the next category of disciplines, about a third of respondents used data collected by someone outside of their research team on a regular basis: Physical Sciences (37.1%), Geology/Earth Science (33.1%), Information/Computer Science (32.6%), Environmental Science/Ecology (32.2%), and Agriculture and Natural Resources (27.7%). In the last category, less than a quarter of respondents were regular users of others’ data, including Engineering (23.6%), Other (22.0%), Biology (16.7%) and Psychology (13.6%).

#### Barriers to data sharing

**Fig 9:**
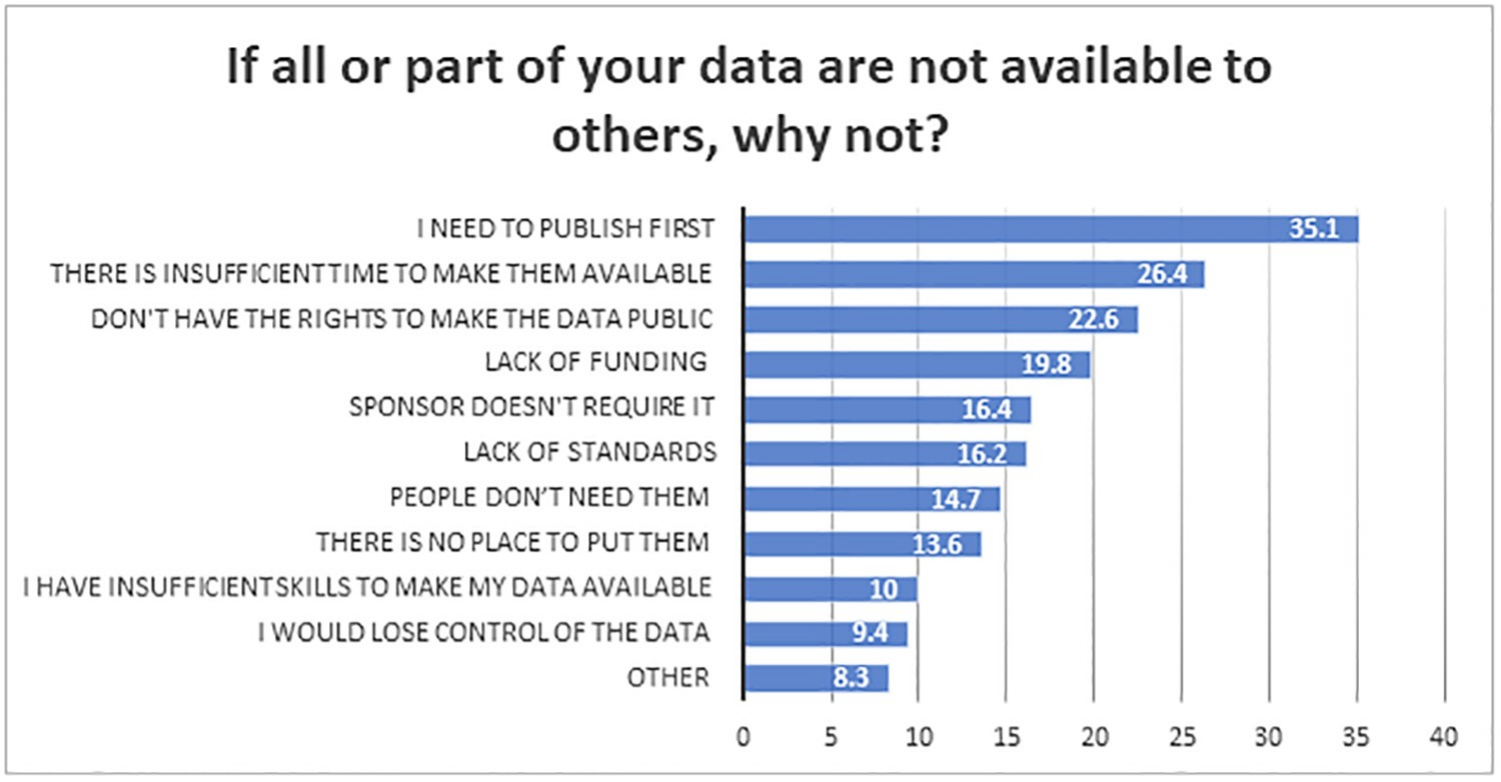
“If all or part of your data are not available to others, why not (Choose all that apply)?” (n = 2184).

A multiple-response question asked researchers to select from a list of possible reasons why all or part of their data might not be available to others. For those who acknowledged that at least some of their data were not available, the reasons most commonly selected included the need to publish first; insufficient time to make the data available; lack of rights to make the data public; and the lack of funding (Fig 9). These barriers to data sharing are similar to those observed in earlier studies [5, 6, 7]

#### Metadata

**Fig 10:**
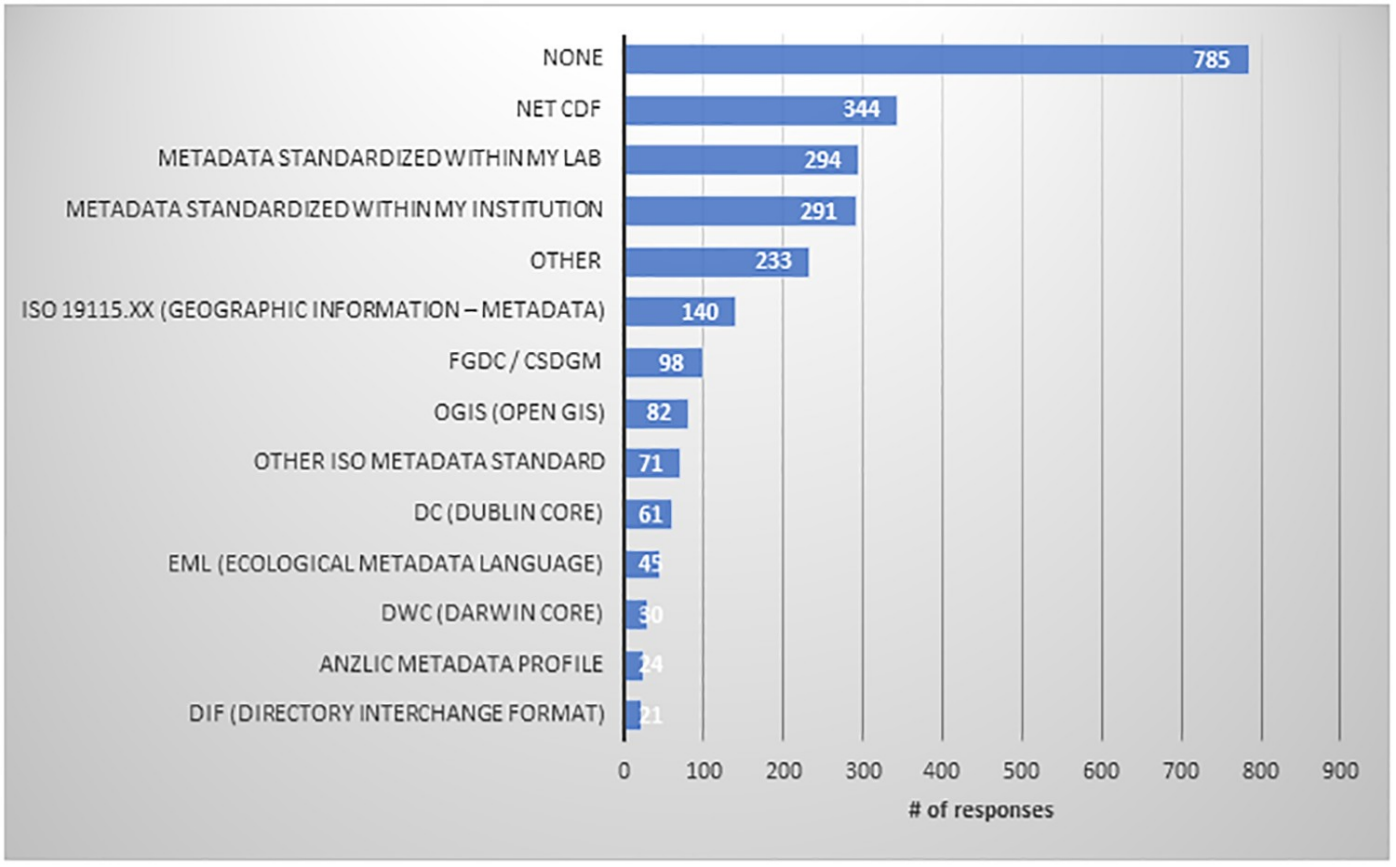
“What metadata standards do you currently use to describe your data, if any? (Choose all that apply)”.

More than a third of respondents (36.4%) said they use some metadata standard to describe their data. The survey instrument presented a list of many commonly known metadata standards–with an addition of an “Other” option, and asked them to identify any that they used; responses are listed in Fig 10. Significantly, almost an equal number indicated they use no metadata standard (“None”) (35.9%), and nearly a quarter said they use "Metadata standardized within my institution/lab" (24%).

The use of a metadata standard differed significantly by sector of employment (Chi-Square = 53.532; p = .000). Government employees are most likely to use some metadata standard (54.9%), followed by respondents working in the commercial sector (43.4%). In the academic, non-profit and other sectors, only about a third of respondents were using a specified metadata standard.

#### Institutional framework

Ensuring good practices in data management is an increasingly important priority for many funding agencies [37, 38, 39]. Almost half of the respondents (48.6%) reported that their primary funding agency requires a data management plan, while 13.2% are not sure if a data management plan were required or not.

Significantly, some disciplines appear to be much more likely to require data management plans from their scientists (Chi-Square value = 63.41; p = 0.000). Respondents representing Space and Planetary Science ranked number one in being required by their primary agency to provide a data management plan (71.4%), followed closely by Marine/Ocean Sciences (64.1%). For all results based on subject discipline, see Table 10.

**Table 10:** Crosstab between “primary funding agency requiring data management plan” and subject discipline. (n = 1966).

Discipline	Primary funding agency requiring data management plan: yes n (%)	Primary funding agency requiring data management plan: no n (%)	Primary funding agency requiring data management plan: don’t know n (%)
Agriculture and Natural Resources	35 (39.3%)	47 (52.5%)	7 (7.9%)
Atmospheric Science	107 (48.4%)	94 (42.5%)	20 9.0%)
Biology	46 (41.1%)	39 (34.8%)	27 24.1%)
Information/Computer science	42 (54.5%)	26 (33.8%)	9 (11.7%)
Environmental Science/Ecology	158 (52.0%)	107 (35.2%)	39 (12.8%)
Engineering	50 (36.2%)	71 (51.4%)	17 (12.3%)
Geology/Earth Science	178 (52.4%)	121 (35.6%)	41 (12.1%)
Hydrology	55 (52.9%)	38 (36.5%)	11 (10.6%)
Physical Sciences	109 (51.2%)	71 (33.3%)	33 (15.5%)
Psychology	10 (45.5%)	11 (50.0%)	1 (4.5%)
Other	122 (44.9%)	105 (38.6%)	45 (16.5%)
Marine/Ocean	25 (64.1%)	9 (23.1%)	5 (12.8%)
Space and Planetary Science	15 (71.4%)	4 (19.0%)	2 (9.5%)

Over a third of respondents (36.9%) said that their organization had a formal process for managing data in the short term, while almost half of the respondents (45.0%) said their organization did not have one. The situation is very similar with long-term data storage: 38.2% report that their organization had a formal process for storing data beyond the life of the project, while 39.9% did not have such a process.

Responses indicate significant differences among work sectors in the organization’s involvement in management and data storage. Non-profit (62.3%) and commercial (60.7%) sectors are leaders in short-term data management (Pearson Chi-Square = 93.106, p = .000), followed by the government sector (52.5%). According to respondents, academia is the least involved at an organizational level in short-term data management: only about a third of respondents who work in an academic setting say that their organizations have a formal process for data management. With respect to establishing processes for long-term data storage, the government sector is leading (61.8%), followed by commercial (54.1%) and non-profit (51.4%). Again, the academic sector seemed to be the least involved in processes addressing long-term data storage, with only 31.3% of respondents stating that their employers have a formal process for storing data beyond the life of the project. Those respondents whose organizations have a formal process for short-term data management or long-term data storage report that the following actors/units were involved in data management (Table 11).

**Table 11:** “You have expressed agreement that your organization or project has a formal process for managing or storing data during or beyond the life of the project (short-term or long-term). Which of the following are involved with this process? (Choose all that apply)”.

Answer	n (%)
Information technology support unit(s) (e.g., Office of Information Technology, IT Support Center)	392 (18.4%)
Colleagues in my own unit/department	383 (18%)
Designated data manager(s)	364 (17%)
Research support unit(s) (e.g., Office of Research, Office of Sponsored Programs and Contracts)	359 (16.9%)
The library	255 (12%)
Administrative office(s) (e.g., Department Heads, Deans, Provosts, Program Offices, Research Offices, Divisions, Directorates / Directors, Managers)	181 (8.5%)
Other	55 (2.6%)

Responses indicated a fairly even distribution of responsibility for data management and storage activities across IT units, colleagues, and data managers. There are some differences in what units/actors are involved in managing or storing data during or beyond the life of the project by the respondent’s primary sector of employment (Table 12). For example, in the academic sector, most respondents named “Research support unit(s)” (15.8%), followed by “Colleagues in my own unit/department (14.8%)”, and “IT units” (14.5%). In the Government sector, data management and storage appear to be more structured and organized: the most popular option among respondents employed by governments indicates allocation of responsibilities to "Designated data managers" (40.2%), followed by “IT departments” (32.9%) and “Colleagues in my own unit/department” (30.9%) and “Research support unit(s)” (24.6%). In Commercial section, the most popular answers are “IT units” (31.6%), “Colleagues in my own unit/department” (30.3%), and “Designated data manager(s)” (25.0%).

**Table 12:** Crosstab between a primary sector and "which of the following are involved in data management/storage in your organization?”.

Actors/Units	Academic	Government	Commercial	Non-profit
Research support unit(s)	241 (15.8%)	85 (24.6%)	11 (14.5%)	16 (18.0%)
The library	193 (12.7%)	43 (12.4%)	9 (11.8%)	8 (9.0%)
Information technology support unit(s)	221 (14.5%)	114 (32.9%)	24 (31.6%)	25 (28.1%)
Administrative office(s)	102 (6.7%)	55 (15.9%)	9 (11.8%)	10 (11.2%)
Designated data manager(s)	175 (11.5%)	139 (40.2%)	19 (25.0%)	21 (23.6%)
Colleagues in my own unit/department	225 (14.8%)	107 (30.9%)	23 (30.3%)	19 (21.3%)
Other	31 (2.0%)	16 (4.6%)	3 (3.9%)	4 (4.5%)

#### Limitations

The two-wave distribution method led to data being gathered over nine months, which is not optimal but which still provides value since it fell within one academic year. We cannot calculate a total response rate for the second wave of the survey because it was distributed to professional organizations and individual researchers and we do not know how many people received or saw the invitation for the survey.

Due to the IRB requirements, the respondents could skip any question or stop the survey at any time, so the number of responses to each question may differ. The length of the survey, estimated to take about twenty minutes to complete, could have potentially contributed to survey fatigue. In addition, since we rely on a volunteer sample, self-selection bias may have occurred, stemming from the fact that people who were knowledgeable and had an opinion about data sharing could have been more likely to take the survey or conversely, they may have felt that there was no need to answer this survey since these practices are ingrained in their work habits. Since the results of the survey were self-reported, we assume that participants responded truthfully and to the best of their ability.

## Discussion and conclusions

### Data use/data storage: Researchers store their data in a variety of places, representing good, mediocre and bad data practices

Most researchers report they store their data on their personal computer (60.3%), departmental servers (42.9%) or USB drives (29.8%), but storage options associated with good data practices are also being used to a lesser extent. Among these, the most popular option is other data repository or archive (16.6%), institution’s repository (16.5%), discipline-based repository (5.6%), and publisher or publisher related-depositories (4.6%). Governmental and non-profit employees seem to be the leaders in good data practices, while academics and employees of the commercial sector are lagging behind. Age seems to have an inverse pattern; adhering to good data storage practices increased in each age group, starting from 19.2% in the youngest age group, to 31.1% in the oldest category.

Respondents appear to be satisfied with short-term storage solutions that provide them with more physical proximity to their data during the collection and analysis phases of their project; however, only half of them are satisfied with available mechanisms for storing data beyond the life of the project. If data resources are to be stored and preserved for the long-term, organizations need to provide easier access to long-term data storage solutions, and training and assistance to researchers on long-term data management.

Survey responses indicate that the available data management assistance to researchers is often inadequate or not known to them. Respondents in Information Science, Engineering, and Agriculture/Natural Resources disciplines appear to be more cognizant of available resources and engage them most frequently to support their data management needs. Approximately a third of respondents from these three disciplines say they consult with data managers or librarians; significantly, responses from researchers in all other disciplines indicate low rates of engagement—between ten and twenty percent—of professionals to assist with their data management needs. Further research would be necessary to determine if these distinctions are the result of broader availability of data management services in specific disciplines, or if the researchers in certain disciplines are simply more aware of the existence of such services.

Researchers employed in the government sector are most likely to work with data managers, perhaps because of open data mandates and requirements for adherence to established data management practices in government agencies. Somewhat confoundingly, data from this survey indicates that researchers in the academic sector are least likely to ask data managers or librarians for assistance in finding data for their research, even as they are the sector most likely to have these institutionalized services available to them. Although librarians and data managers may be available, most researchers in academia are unaware of assistance or do not take advantage of such assistance.

### Data reuse, sharing and barriers

The vast majority of respondents have a positive attitude towards sharing data and data reuse. Over a third of respondents use data collected by others in their research on a regular basis (most of those respondents representing governmental and commercial sections), while 87% would use data collected by someone else if it could be easily accessed. According to respondents, written details on collection and quality assurance methods, explicitly stated metadata standards and detailed provenance information are the most important criteria influencing their confidence in using data collected by others.

The idea of data sharing is seen in a positive light, with 86.7% saying they would be willing to share their data across a broad group of researchers. Respondents would be more willing to share if they could place some conditions on use (56.4%) on those reusing their data. A citation is an almost universal requirement: 92.1% of respondents said it was important for them to receive citation credit by those who would use their data. The need to publish first was reported as the main barrier to sharing data, followed by the lack of rights to data, time to properly prepare data for sharing, and funding restrictions limiting their ability to prepare and deposit the data.

A vast majority of respondents feel that the lack of access to data generated by other researchers or institutions was a major impediment to progress in science at large, yet only about a half of them thought that it restricted their own ability to answer scientific questions. This discrepancy between the perceived effects on others and the respondents themselves could be viewed as a variation of the third person effect theory and could be further explored in future research.

### Institutional framework/data management support

Data management plans are now required by funding agencies in many countries [37, 38]. Around half of the respondents say their funders require a data management plan, most of them in the US and Europe; within research communities, the Space and Planetary Science and the Marine Science fields appear to be strong proponents of data management plans. Regarding a formal process for data management, non-profit and commercial sectors are leading (with over 60% of respondents indicating that their organizations have a formal process), while the academic sector seemed to be least likely to have institutionalized processes. Designated data managers and data librarians can promulgate awareness of requirements and assist researchers in preparing good data management plans.

Across all sectors, respondents perceive libraries as less involved than other units in their organizations in providing data management support services. Either many libraries are not providing these services, or their existing services are not appropriately communicated to researchers. Employees of government institutions indicate stronger support for organizing data management and storage by designated professional employees, including data managers, research support units, and IT units. Responses from the government sector indicated the highest levels of available data management assistance and training to employees, while respondents from the academic sector observed the lowest rates of available training and assistance. Clearly there is a need to do more training and assistance in the academic sector, or to more fully communicate the availability of existing services.

Satisfaction with the tools and practices associated with data management seems to be low: only about a third of respondents’ express satisfaction with tools for preparing metadata and their ability to track and verify provenance information. Access to appropriate repositories also seems to be an issue: only 37.4% of respondents say it is easy for them to locate a suitable repository for deposit of data.

Slightly more than a third of respondents use some kind of commonly recognized metadata standard, while another third say they use any standard, and about a quarter say they use a “metadata standardized within my institution/lab.” The government and commercial sectors are leaders in using a metadata standard, with over half of government respondents reporting use of such standards. Low rates of uptake of widely used metadata standards is a concern, both for data discovery and for the necessary levels of description that are vital to understanding [39]. Data managers, data librarians, and affordable metadata tools should help researchers with metadata creation. It is unrealistic to expect every researcher to be a metadata expert, but search and retrieval of data sets rely on complete and accurate metadata, and understanding of data for responsible reuse requires comprehensive details about methodologies, data structure and processing, and provenance. There is more work to be done in making this happen.

## Conclusions

The results of this study align with other research projects that examine the attitudes and behavior of scientists in regards to the use, storage, and reuse of data. For example, our results show similar trends that were discovered by researchers from Leiden University’s Centre for Science and Technology Studies and Elsevier, who used a combination of qualitative and quantitative methods to examine motivations and barriers of data sharing by researchers [40]. The results of our survey have similar results, highlighting the existing gap between positive attitudes towards open data and open science with researchers actually implementing good data practices. Both surveys also demonstrated higher levels of good data practices by researchers working in scientific fields that do not deal with human subjects and where a significant amount of data is gathered by large-scale instrumentation shared by a number of researchers and research teams.

Since most questions of this survey closely emulated those from two previous studies conducted in 2011 and 2015 by DataONE Usability and Assessment group [5, 6], we are able to discuss the general direction of the changes in attitudes and behaviors of the global scientific community. The progress in moving towards open science is reflected in the growing acceptance of the concepts crucial for open science, specifically the concepts of data sharing and reuse. Over the past decade, the number of respondents who said that they are willing to share their data with other researchers has increased. At the same time, the results of the survey demonstrate a growing acceptance of data reuse.

In addition to the increase in the number of scientists who hold positive attitudes towards data sharing and reuse, there is also a change in behavior: more respondents display good data practices, reuse data, and share their data. The use and understanding of metadata is another indicator of the progress that a scientific community is making in accepting and following good data practices. Similarly, the number of respondents who acknowledge various barriers that prevent them from sharing their data has been steadily decreasing over the examined time frame.

Over three waves of the survey, there is a noticeable positive dynamic in terms of organizational involvement in data sharing, use, and reuse. The number of respondents who stated that their organizations require data management plans is on the rise, and more say their organizations offer training and assistance in issues related to data management. Although the reasons behind this dynamic are outside of the framework of this study, we can hypothesize that general acceptance of the importance of open data and open science by various stakeholders in the research process, including publishers, governments, and funding agencies have a positive effect on organizations being more involved and more helpful with data management.

Finally, it is important to acknowledge the differences in data sharing, use, and reuse between respondents employed by four work sectors. The governmental sector emerged as a leader in positive attitudes, good data practices, as well as organizational involvement in data training and management. Examination of several important indicators, such as willingness to share and reuse data, displaying good data practices, and growing organizational involvement in data management show the governmental sector to be the frontrunner in accepting and implementing data management practices.

The results of this study can provide additional insights into what aspects of the implementation of FAIR principles to promote open data require extra attention and effort from stakeholders. For example, ensuring best data practices through Data Management Plans is listed among fifteen priority recommendations by the European Commission [23], but only about half of the respondents currently indicate that their primary funding agency requires a data management plan.

Our international study of the data management practices and attitudes of scientists shows there is variation in data practices based on the work sector, subject discipline, and, sometimes by age of researchers. Although attitudes towards data sharing and data use and reuse are mostly positive, practice does not always support data storage, sharing, and use in the future. While there is noticeable progress in moving toward open data and open science, there still is a discrepancy between positive attitudes and actual implementation of those principles by the scientific community. Goodwill and positive attitudes, however, suggest that with stronger organizational involvement in providing training and support of good data practices, there is potential for major positive changes. Assistance from data managers or data librarians, readily available data repositories for both long-term and short-term storage and educational programs to engender good data practices are clearly needed.

## Supporting information

S1 Table Primary subject discipline. (DOCX)

S2 Table (DOCX)
