# Supplementary material for: Data sharing, management, use, and reuse: Practices and perceptions of scientists worldwide
Source: PLoS One. 2020 Mar 11;15(3):e0229003. doi: 10.1371/journal.pone.0229003 (PMC7065823; doi:10.1371/journal.pone.0229003)
Supplement: S1 Table — (DOCX) [file pone.0229003.s001.docx]

Table 1. Primary subject discipline.

| **Discipline** | **%** |
| --- | --- |
| Geology/Earth Science | 17.0 |
| Environmental Science/Ecology | 15.5 |
| Other | 15.3 |
| Atmospheric science | 11.1 |
| Physical sciences | 10.7 |
| Engineering | 6.9 |
| Biology | 5.4 |
| Hydrology | 5.2 |
| Agriculture and Natural Resources | 4.5 |
| Information/Computer science | 4.2 |
| Marine/Ocean | 2.0 |
| Psychology | 1.0 |
| Space and Planetary Science | 1.0 |
| Law | .0 |
| Total | 2098 |
