# Supplementary material for: Data sharing, management, use, and reuse: Practices and perceptions of scientists worldwide
Source: PLoS One. 2020 Mar 11;15(3):e0229003. doi: 10.1371/journal.pone.0229003 (PMC7065823; doi:10.1371/journal.pone.0229003)
Supplement: S2 Table — (DOCX) [file pone.0229003.s002.docx]

Table 2.

| Barrier | % | n |
| --- | --- | --- |
| I need to publish first | 35.1 | n=767 |
| There is insufficient time to make them available | 26.4 | n=578 |
| Don't have the rights to make the data public | 22.6 | n=495 |
| Lack of funding | 19.8 | n=433 |
| Sponsor doesn't require it | 16.4 | n=359 |
| Lack of standards | 16.2 | n=354 |
| People don’t need them | 14.7 | n=323 |
| There is no place to put them | 13.6 | n=299 |
| I have insufficient skills to make my data available | 10 | n=219 |
| I would lose control of the data | 9.4 | n=206 |
| Other | 8.3 | n=182 |
